# Combination photodynamic therapy and bevacizumab for choroidal neovascularization associated with toxoplasmosis

**DOI:** 10.4103/0301-4738.73728

**Published:** 2011

**Authors:** Pukhraj Rishi, Anusha Venkataraman, Ekta Rishi

**Affiliations:** Shri Bhagwan Mahavir Vitreoretinal Services, Sankara Nethralaya, 18, College Road, Chennai - 600 006, Tamil Nadu, India

**Keywords:** Bevacizumab, choroidal neovascularization, photodynamic therapy, toxoplasmosis

## Abstract

A 14-year-old girl presenting with visual loss in both eyes was diagnosed to have healed toxoplasma retinochoroiditis in the right eye with active choroidal neovascularization (CNV) secondary to toxoplasmosis in the left. She underwent combination photodynamic therapy (PDT) and intravitreal bevacizumab as primary treatment. PDT was performed as per the ‘Treatment of Age-related Macular Degeneration by Photodynamic therapy’ study protocol and was followed by intravitreal bevacizumab after 2 days. CNV regressed at 8 weeks of follow-up and remained stable at 8 months of follow-up. The initial visual acuity improved from 20/120 to 20/30. Combination therapy with PDT and intravitreal bevacizumab appears to be effective in the treatment of CNV secondary to toxoplasma retinochoroiditis.

Ocular toxoplasmosis is an important cause of choroidal neovascularization (CNV) in the pediatric age group.[1] The development of CNV adjacent to retinochoroidal scars is a well-known late complication contributing to loss of useful vision due to foveal involvement. Treatment modalities for CNV secondary to toxoplasma retinochoroiditis include laser photocoagulation, submacular surgery, photodynamic therapy (PDT) and anti-vascular endothelial growth factor (VEGF) agents.[1] We report a case of CNV secondary to ocular toxoplasmosis treated with a combination of verteporfin–PDT and intravitreal bevacizumab. Combination therapy for CNV secondary to toxoplasmosis has not been reported in the past.

## Case Report

**Figure 1:**
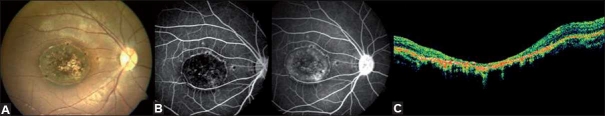
At presentation, right eye color fundus photograph (A) reveals a punched-out pigmented lesion with well-demarcated borders at the macula suggestive of a healed toxoplasma scar. Fluorescein angiography (B) reveals staining of the retinal scar. Optical coherence tomography reveals retinal atrophy corresponding to the lesion (C)

**Figure 2:**
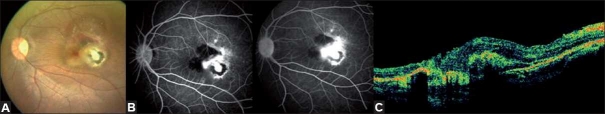
At presentation, left eye color fundus photograph (A) reveals a pigmented lesion at the macula along with a choroidal neovascular membrane, adjacent subretinal hemorrhage and fluid medial to the pigmented scar. Fluorescein angiography (B, C) reveals an active, subfoveal classic choroidal neovascular membrane with profuse leakage. Blocked choroidal fluorescence due to the overlying hemorrhage is also noted. Optical coherence tomography reveals subfoveal choroidal neovascular membrane and subretinal fluid adjacent to a subretinal scar

A 14-year-old girl presented with a 1-year history of deterioration of vision in both eyes. There were no significant ocular problems in the past. Medical and family history and systemic examination were unremarkable. On ocular examination, her best corrected visual acuity (BCVA) at presentation was CF 1 mt, N36 in the right eye and 20/120, N18 in the left eye. Biomicroscopic examination did not show any evidence of intra-ocular inflammation and the anterior segment was unremarkable in both eyes. Fundus evaluation of the right eye showed the presence of a punched-out pigmented lesion with well-demarcated borders at the macula that was suggestive of a healed toxoplasma lesion [Fig. 1A]. Clinical findings were confirmed on fluorescein angiography [Fig. 1B] and Optical coherence tomography [Fig. 1C]. Left eye examination not only showed a punched-out pigmented lesion at the macula but also a choroidal neovascular membrane with adjacent subretinal fluid and hemorrhage just medial to the pigmented scar [Fig. 2A].

The clinical findings were confirmed on fundus fluorescein angiography (FFA) and optical coherence tomography (OCT). The FFA of the left eye revealed an active subfoveal classic choroidal neovascular membrane with profuse leakage. Blocked choroidal fluorescence due to the overlying hemorrhage was also noted [Fig. 2B]. OCT revealed subfoveal choroidal neovascular membrane (CNVM) and subretinal fluid adjacent to a subretinal scar [Fig. 2C]. The routine hemogram, kidney function tests and liver function tests were within the normal range. Mantoux test was negative and chest X-ray was normal. The anti-toxoplasma IgM titer was negative; IgG titer was however equivocal. A diagnosis of healed toxoplasma retinochoroiditis in the right eye and subfoveal CNVM secondary to toxoplasma retinochoroiditis in the left eye was arrived at. The nature of the disease and treatment options were explained. The patient underwent verteporfin-PDT as per the ‘Treatment of Age-related Macular Degeneration by Photodynamic therapy’ protocol.[2] This was followed by an intravitreal injection of bevacizumab (1.25 mg/0.05 ml) after 2 days.

**Figure 3:**
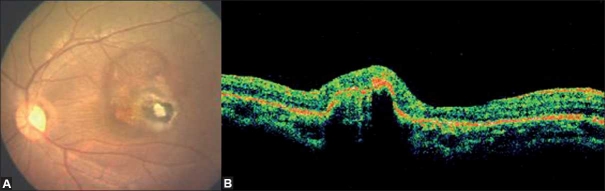
At 8 months of follow-up, color fundus photograph (A) reveals a regressed choroidal neovascular membrane. Optical coherence tomography reveals (B) a high reflective subfoveal scar

At 8 weeks follow-up, the CNV showed regression with no evidence of subretinal fluid or hemorrhage on clinical examination or OCT. The BCVA improved to 20/30, N6 and the fundus remained stable up to the eighth month of follow-up [Fig. 3]. No adverse ocular or systemic event related to the treatment procedure was encountered.

## Discussion

The natural history of CNV secondary to toxoplasmosis reveals a poor visual prognosis.[3–5] The CNV originates adjacent to the retinochoroidal scars and leads to central visual loss due to foveal involvement. The different treatment modalities described for CNV secondary to toxoplasmosis in children include laser photocoagulation, submacular surgery, PDT and anti-VEGF agents.[1] Laser photocoagulation is no longer preferred as it results in a permanent scotoma. Uemura *et al*.[6] assessed the efficacy of surgical removal of membranes in a series of 14 children with subfoveal CNV of which one was secondary to toxoplasmosis. The initial visual acuity of 20/100 in this patient improved to a final visual acuity of 20/20 at 27 months follow-up. The favorable outcome is attributed to the solitary subretinal site of ingrowth that is easily amenable to surgery.[7] However, surgery can have its own complications and does not prevent recurrences.

The use of verteporfin–PDT, although initially used for the treatment of age-related macular degeneration and myopic CNV, has now been extended beyond these indications to inflammatory and infectious etiologies. Mauget Faysse *et al*.[8] have found verteporfin–PDT to be efficacious for subfoveal CNV associated with toxoplasma retinochoroiditis. In their study of eight patients, the mean visual acuity improved from 20/225 to 20/123 during a mean follow-up period of 25 months. Ben Yahia *et al*.[9] recently reported the resolution of two CNVMs secondary to toxoplasma retinochoroiditis following administration of a single intravitreal injection of bevacizumab as primary treatment or as rescue treatment following unsuccessful PDT.

There is however no report of any patient with CNV secondary to toxoplasmosis who has been managed with combination PDT and intravitreal bevacizumab as a primary treatment. The combined regime is postulated to have a beneficial synergistic effect that could reduce the need for repeated injections. Combination therapy using PDT and bevacizumab as the first-line management in such cases could be a viable option.[10] Larger studies with longer follow-up may reveal that visual outcome with combination therapy could be better than PDT alone.
